# Radiotherapy for lentigo maligna and lentigo maligna melanoma – a systematic review

**DOI:** 10.1186/s13014-020-01615-2

**Published:** 2020-07-14

**Authors:** Alexandra Hendrickx, Antonio Cozzio, Ludwig Plasswilm, Cédric M. Panje

**Affiliations:** 1 University of Bern, Bern, Switzerland; 2 Department of Dermatology, Venerology and Allergology, Kantonsspital St. Gallen, St. Gallen, Switzerland; 3 Department of Radiation Oncology, Kantonsspital St. Gallen, Rorschacherstrasse 95, 9007 St. Gallen, Switzerland

**Keywords:** Lentigo maligna, Lentigo maligna melanoma, Radiotherapy, Grenz ray, Superficial X-ray

## Abstract

Lentigo maligna (LM) is the most common subtype of in situ melanoma und occurs frequently in the sun-exposed head and neck region in elderly patients. The therapeutic “gold standard” is surgical excision, as there is the risk of progression to invasive (lentigo maligna) melanoma (LMM). However, surgery is not feasible in certain patients due to age, comorbidities or patient preference. Radiotherapy using Grenz rays or superficial X-rays has been established as non-invasive alternative for the treatment of LM and LMM. We performed a systematic literature search of MEDLINE and Embase databases in September 2019 and identified 14 patient series using radiotherapy for LM or LMM. No prospective trials were found. The 14 studies reported a total of 1243 lesions (1075 LM and 168 LMM) treated with radiotherapy. Local recurrence rates ranged from 0 to 31% and were comparable to surgical series in most of the reports on radiotherapy. Superficial radiotherapy was prescribed in 5–23 fractions with a total dose of 35–57 Gy. Grenz ray therapy was prescribed in 42–160 Gy in 3–13 fractions with single doses up to 20 Gy. Cosmetic results were reported as “good” to “excellent” for the majority of patients.

In conclusion, the available low-level evidence suggests that radiotherapy may be a safe and effective treatment for LM and LMM. Data from prospective trials such as the phase 3 RADICAL trial are needed to confirm these promising findings and to compare radiotherapy to other non-surgical therapies and to surgery.

## Introduction

Lentigo maligna (LM) is the most prevalent melanoma in situ subtype which generally occurs in the sun-exposed skin of the head and neck region in elderly, fair-skinned persons [1, 2]. Although LM is a pre-invasive intraepidermal melanocytic malignancy, case series suggest that there is a lifetime risk for progression to invasive disease in up to 50% [1]. Lentigo maligna melanoma (LMM) is the third most common subtype of invasive melanoma and is defined as the invasive progression of LM [2].

The therapeutic standard for LM is surgical resection with Mohs micrographic surgery [3] or wide local excision with a margin ranging from 5 to 10 mm, but high-quality data on the optimal technique is lacking [4, 5]. For LMM, surgical resection with a margin of 10 to 20 mm with or without sentinel lymph node biopsy depending on tumor thickness are recommended as for other subtypes of invasive melanoma [4].

Radiotherapy has been used as a treatment option for LM, and less commonly for LMM, in patients which are not amenable to surgery [1, 4, 6, 7]. Several patient series have shown that radiotherapy using Grenz rays or superficial X-rays can provide similar local control rates as surgery and improved outcome compared to other non-surgical techniques such as laser therapy or topical immunomodulatory therapy with imiquimod [6].

Although radiotherapy for LM has already been introduced in the 1950ies by Miescher at al. [8], there is only limited evidence available on treatment outcome and the optimal choice of radiotherapy technique [5]. Additionally, many radiation oncologists are not familiar with the treatment of LM and LMM, as superficial irradiation of skin neoplasms is done by dermatologists in many institutions [7].

The aim of our study was therefore to perform a systematic review of the literature on radiotherapy for LM and LMM and to discuss the findings from a multidisciplinary perspective.

## Methods

We performed a systematic literature search based on the recommendations of the PRISMA statement for systematic reviews [9]. We searched the databases MEDLINE and EMBASE using the search terms “lentigo” or “lentigo maligna” or “melanotic precancerosis” or “in situ melanoma” or “Hutchinson” and “radiotherapy” or “grenz ray” or “x-ray” or “roentgen” or “irradiation”.

Studies published in the last 50 years (1970 until September 2019) were included into our analysis. The search was limited to original articles as well as conference abstracts/papers in English or German. Case reports, letters, editorials, book chapters and reviews were excluded from the analysis. Also, we reviewed reference lists from included studies as well as from previously published reviews to ensure a complete coverage of the published literature.

The primary outcome reported in our systematic review was local recurrence rate after radiotherapy, secondary outcomes were complete response rate and late skin toxicity.

Publications were included when the following criteria were met:

1. Patient series of clinically or histologically confirmed lentigo maligna (LM) or lentigo maligna melanoma (LMM)
2. Lesions treated by radiotherapy
3. Reported follow-up duration
4. Reported endpoints such as recurrence rate, local control and/or complete response rate

The study selection and review of the included studies as well as the definitive approval of the reviewed data was performed independently by two authors (A.H. and C.P.). Diverging results were discussed with the co-authors. All patient series were included, but no single case reports. In case of overlapping patient series from the same institution, we excluded any article whose patient group was completely included in a more recent publication. When different treatment modalities were reported in a single publication, we only extracted data on radiotherapy.

We extracted the following data from the included studies: year of publication, included period, study design, sample size, local control, complete response rate, follow-up period, determination of clearance, cosmetic results, RT dose prescription and fractionation, and RT techniques including safety margin. Dose prescriptions were listed in Gray (Gy) for contemporary publications and in Roentgen (R) according to the former standard in earlier publications.

## Results

**Fig. 1:**
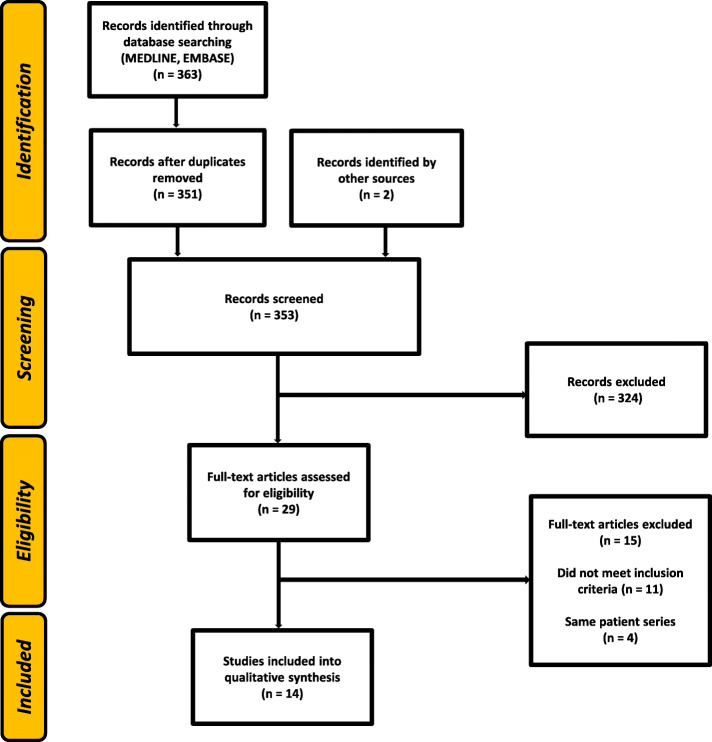
Systematic literature search flow diagram

Our literature search identified 361 records by database search and 2 records by other sources (reference list of reviews or original articles, see Fig. 1). After removal of duplicates, 353 articles were screened by title and abstract review and 324 articles were excluded. Full-text assessment for eligibility was done for 29 articles, and 15 articles were excluded. Eleven articles did not meet the predefined inclusion criteria, four articles were excluded as they reported on the same patient population as more recent publications.

Overall, we identified 14 retrospective series with a total of 1243 lesions (range 5–593 per study) treated from 1941 to 2014 at 10 different institutions in Europe and North America. All included studies are summarized in Table 1.

**Table 1:** Outcome of retrospective studies on radiotherapy for lentigo maligna and lentigo maligna melanoma

First author	Year	Center	Period	Sample size	Local recurrence rate	Complete response rate	Follow-up duration	Cosmetic results	Notes
**Lazarevic** [10]	2019	Zurich, Switzerland	2009–2014	27 LM, 18 M	15% (LM), 17% (M)	n.a.	51 months (mean)	«good»	periocular lesions, 44% of melanoma patients with immunosuppression, 11% LM
**Lamoureux** [11]	2018	Bordeaux, France	2007–2017	42 LM, 6 LMM, 13 LMM with previous surgery	10% (overall), 7% (only LM)	n.a.	22 months (mean), range 3–70 months	9.8% hypopigmentation, 8.1% teleangiectasia, no fibrosis	Conference abstract only
**Hedblad** [12]	2012	Stockholm, Sweden	1990–2009	593 LM or early LMM; 59% primary RT; 12% after partial excision, 29% adjuvant RT after complete excision	Overall 12% (17% primary RT, 9% RT after partial excision, 3% RT after complete excision)	88% (primary RT); 90% (RT after partial excision); 97% (RT after complete excision)	425 patients for at least 24 months, 241 for more than 60 months	«excellent» (15% hypopigmentation, 20% hyperpigmentation, «some» teleangiectasia)	86% facial localization
**Lee** [13]	2011	London, Ontario, Canada	1991–2005	31 LM	29% (LM)	n.a.	46.3 months (median)	19.4% teleangiectasia, 6.5% hypopigmentation	89.3% head and neck region
**Zalaudek** [14]	2003	Graz, Austria	1990–2000	15 LM	13% (LM)	n.a.	5-year recurrence rate reported	n.a.	All lesions in head and neck region
**Farshad** [15]	2002	Zurich, Switzerland	1950–2000	93 LM, 54 LMM, 3 LM and LMM (more than one lesion in 4%)	7% overall, (for 101 patients with a follow-up of at least 2 years)	n.a.	8 years (mean)	«acceptable»	90% facial localization
**Schmid-Wendtner** [16]	2000	Munich, Germany	1987–1998	42 LM, 22 LMM	0% (LM), 9% (LMM)	n.a.	15 months (median, range 1–96)	“good or excellent”, no ulcers or fibrosis	98% head and neck region
**Christie** [17]	1996	Westmead, Australia	1989–1995	5 LM	0% (LM)	100% (LM)	16 months (median, range 8–37 months	«favourable», teleangiectasia in 2 patients	All lesions in head and neck region
**Tsang** [18]	1994	Toronto, Ontario, Canada	1968–1988	36 LM	11% (LM)	n.a.	72 months (median)	«acceptable», «poor» in 11% (skin pallor, atrophy, teleangiectasia)	18 patients already included in previous publication [19]; 92% head and neck area
**Harwood** [19]	1983	Toronto, Ontario, Canada	1958–1982	23 LM, 28 LMM	10% (LM), 8% (LMM)	n.a.	LM: (26 months (median, range 5–96 months); LMM: 24 months (median, range 6–96 months)	n.a.	96% head and neck region
**Richter** [20]	1977	Dresden, Germany	1957–1975	12 LM, 5 LMM	0%	100%	12–84 months	“excellent”	79% head and neck region; publication in German
**Kopf** [21]	1976	New York, USA	1964–1973	16 LM	31%	94%	median: 32.5 months (range 6–113)	“fair to excellent” in most cases	
**Braun-Falco** [22]	1975	Munich, Germany	1955–1970	68 LM	0% (LM)	100% (LM)	36 months (mean, range 12–120 months)	«good»	86% facial or head area; histological diagnosis only in 10 cases; publication in German
**Arma-Szlachcic** [23]	1970	Zurich, Switzerland	1941–1965	69 LM, 19 LMM	3% (LM), 0% (LMM, but metastatic spread in 26%)	100% (LM)	at least 60 months in 74 patients	n.a.	84% facial localization; publication in German

*LM* Lentigo maligna; *LMM* Lentigo maligna melanoma; *M* Melanoma (not further specified); *n.a.* Not available; *RT* Radiotherapy

A total of 1075 LM lesions and 168 LMM were treated. The average patient age in the publications (mean or median reported) ranged from 59.2–79.8 years. The vast majority of the irradiated lesions were located in the head and neck area (79–100%). Follow-up ranged from 15 to 96 months (mean or median).

Grenz rays (10–30 kV) were used in 43% of the patient series, 36% used superficial or orthovoltage X-rays (30–250 kV), 21% used both techniques. Technical specifications of radiotherapy are summarized in Table 2.

**Table 2:** Technical parameters of radiotherapy for lentigo maligna and lentigo maligna melanoma

First author	RT techniques	RT dose and fractionation	Margin
**Lazarevic** [10]	Grenz or soft x-rays (10–30 kV)	42–120 Gy in 3–13 fx, 3–4 day intervals	15 mm
**Lamoureux** [11]	Superficial x-rays («contact therapy»), 30–150 kV	40 Gy in 10 fx, 2x/week; or 39 Gy in 13 fx, 3x/week	5–10 mm
**Hedblad** [12]	Grenz rays (10 kV)	100–160 Gy, in 6 fx, 2x/week	10 mm
**Lee** [13]	Superficial x-rays	50 Gy in 20 fx	n.a.
**Zalaudek** [14]	Grenz rays, 10 kV	120 Gy in 6 fx	at least 5 mm
**Farshad** [15]	Grenz rays (12 kV, 107 patients) or superficial X-rays (20 to 50 kV, 57 patients)	12 kV: 100–120 Gy, 10–12 fx, 2x/week; 20–50 kV: 42–54 Gy, 7–9 fx, 2x/week	7–10 mm
**Schmid-Wendter** [16]	Grenz rays (14.5 kV) excision of the nodular part of LMM before RT	100 Gy, 10 fx, 5x/week	5–20 mm
**Christie** [17]	Superficial x-rays (100 kV)	44 Gy in 11 daily fx or 57.5 Gy in 23 daily fx	at least 10 mm
**Tsang** [18]	Superficial/ orthovoltage x-rays (100–250 kV)	35 Gy in 5 daily fx, 45 Gy in 10 daily fx or 50 Gy in 15 daily fractions	5–10 mm
**Harwood** [19]	Superficial/orthovoltage x-rays (LM: 100 kV; LMM: 125–175 kV)	35 Gy in 5 daily fx, 45 Gy in 10 daily fx, 50 Gy in 10–20 daily fx	at least 10 mm
**Richter** [20]	LM: Grenz rays (9 kV) or superficial x-rays (48.5 kV) LMM: superficial x-rays (26–38 kV)	LM: 10,000 R in 5–10 fx LMM: 6000–10,000 R in 12–20 fx	n.a.
**Kopf** [21]	Grenz rays (12 kV)	10,000 R in 5 fx, 3–4 day intervals	5 mm
**Braun-Falco** [22]	Grenz rays (14.5 kV)	10,000 R in 5–10 daily fx	n.a.
**Arma-Szlachcic** [23]	LM: Grenz rays (12 kV); LMM: Grenz rays or superficial x-rays (50–60 kV)	LM: most commonly 10,000–12,000 R in 5–6 fx every 5–7 days; LMM: various schedules	n.a.

*Fx* Radiotherapy fraction; *LM* Lentigo maligna; *LMM* Lentigo maligna melanoma; *M* Melanoma (not further specified); *n.a.* Not available

Dose prescription and fractionation for Grenz rays were 42–160 Gy in 3–13 fractions given daily to every 4 days. For superficial and orthovoltage therapy, dose prescription was 35–57 Gy. Radiotherapy was administered in 5–23 fractions given daily to every 4 days.

Overall, recurrence rates ranged from 0 to 31%. For Grenz rays, several series from European centers reported recurrence rates from 0 to 17% [12, 14, 16, 20, 22, 23], whereas a small study from the USA with 16 patients using Grenz rays reported a recurrence rate of 31% [21]. For superficial radiotherapy and orthovoltage therapy, local recurrence rates ranged from 0 to 29%.

Cosmetic results were reported as “good” to “excellent” for the majority of patients, but late skin changes such as atrophy, teleangiectasia as well as hypopigmentation or hyperpigmentation were reported in up to 20%. However, no radiation ulcers or fibrosis were reported.

## Discussion

Radiotherapy has been used for decades as non-invasive alternative for patients with LM and LMM who are not amenable to surgical resection [7] and is – particularly for LM – listed as a treatment option in current melanoma guidelines [4, 24]. Radiotherapy is typically used in patients where surgery may result in poor functional or cosmetic outcomes or if there is an increased surgical risk due to patient age, comorbidities or medications such as anticoagulation [1]. However, all evidence for the use of radiotherapy to treat LM and LMM comes from retrospective patient series. Our review summarizes all available studies from the last 50 years and found 14 eligible studies.

Reviewing 14 retrospective patient series with treatment of a total of 1243 lesions by either Grenz rays or superficial X-rays, recurrence rates from 0 to 31% were reported. The greater part showed “good” to “excellent” cosmetic outcome, but long-term skin changes were reported in up to 20%.

The majority of eligible studies on radiotherapy for LM and LMM had recurrence rates which are comparable to surgical resection [6] and more favorable than studies on other non-invasive treatments such as laser therapy or topical immunomodulatory therapy with imiquimod [6].

In contrast to previous reviews [5, 6], we have included more recently published patient series [10, 11] as well as publications in German [20, 22, 23] with special focus on radiotherapy techniques and fractionation. Also, we reviewed radiotherapy outcomes for LM as well as LMM, as many publications report on both patient groups [11, 12, 15, 16]. Both patients with LM and LMM admitted for radiotherapy are likely to have similar characteristics such as large overall lesion size (invasive and noninvasive components), advanced age and comorbidities favoring non-surgical therapeutic approaches. The results of the presented patient series confirm that radiotherapy for LMM with or without previous excision of nodular components provides low recurrence rates [11, 15]. Congruently, the 2019 European consensus-based interdisciplinary guideline for malignant melanoma lists radiotherapy as treatment option for inoperable LMM in addition to LM [4].

### Radiation techniques and outcome

Our results show that a wide range of dose prescriptions and fractionations have been used for the treatment of LM and LMM.

In general, treatment techniques can be divided into Grenz ray therapy and superficial radiotherapy. Grenz rays generally have an energy range from about 10 to 30 kV and are at the lower end of the X-ray spectrum. Grenz rays have a very low tissue penetration with a half-dose depth of about 1 mm. Superficial X-rays are generally considered to have an energy range from 30 to 150 kV, but specifications of this range vary a lot. Their half-dose depth can be more than a centimeter. Other available techniques such as megavoltage photons, electrons or brachytherapy are also available for cutaneous tumors such as LM and LMM [1, 25], but have not been used in the reviewed studies. However, brachytherapy moulage techniques [26] or linac-based intensity-modulated radiotherapy [27] may be good therapeutic options particularly in case of extensive scalp lesions.

Due to the low tissue penetration depth, Grenz ray radiotherapy with low beam energies (9–14.5 kV) can be safely performed with total doses of at least 100 Gy (corresponding approximately to 10,000 R in earlier series) with a single fraction dose of 10–20 Gy.

Superficial radiotherapy was more commonly applied with smaller single fractions of 2.5–7 Gy and lower total doses.

A preferred fractionation schedule or a dose-response relationship cannot be identified based on the available studies. For Grenz ray therapy with up to 15 kV photons, a therapeutic regimen with single fraction doses of 10–20 Gy and total doses above 100 Gy, is highly effective and safe, but this regimen should never be translated to higher Grenz ray energies, to superficial X-rays or to other modalities with deeper tissue penetration due to the risk of excessive toxicity. For superficial radiotherapy, biologically equivalent doses to 54–60 Gy in 2 Gy per fraction have been recommended for primary radiotherapy for LM [1]. As protracted normofractionated treatment regimens impose logistical problems on the typically elderly and comorbid patient presenting with LM or LMM, hypofractionated schedules with doses of 2.5–4 Gy per fraction are generally favored in clinical practice, although doses up to 7 Gy have been reported [18].

The radiation field in the treatment of LM and LMM typically includes the visible lesions with a safety margin of 5–20 mm of the surrounding skin to account for microscopic disease extension. Although data is limited, it has been suggested that smaller margins may result in an increased risk of out-of-field recurrences [7]. If anatomically possible, a margin of at least 10 mm from the visible lesion to the field edge is recommended [1]. Additionally, pre-treatment mapping biopsies or in-vivo reflectance confocal microscopy may help to assess the extent of the lesion in selected cases [4].

There have been concerns that Grenz ray therapy with a half-dose depth of approximately 1 mm may provide insufficient dose coverage of LM extending into skin appendages (e.g. hair follicles), skin folds or for clinically inapparent LMM components. However, case series demonstrate that Grenz ray therapy can achieve high local control rates of approximately 90% or more at experienced European centers despite these concerns [10, 12]. In contrast, Grenz ray treatment was abandoned in a US center after a recurrence rate of nearly one third [21] which may be due to too small margins, a half-dose depth of less than 1 mm and potential quality assurance issues [7].

Outside of centers experienced in low energy Grenz ray therapy, we support the opinion of Fogarty et al. [1] to favor superficial radiotherapy or at least higher Grenz ray energies for LM and LMM which allow a complete coverage of the target volume (including skin appendages) with the therapeutic dose. Additionally, superficial X-rays are likely to result in less dose inhomogeneities in skin areas with concave or convex surfaces.

As already stated previously, there is a trend that radiation oncologists prefer superficial RT techniques whereas Grenz ray therapy is more commonly used by dermatologists [7].

Pigmentations have disappeared within one to 24 months after the end of the treatment. It is generally recommended to assess pigmentation clearance 6 months after radiotherapy [1]. Recurrences were reported within three to 108 months which highlights the need of follow-up visits after radiotherapy.

### Cosmetic results

Overall, most series report favorable cosmetic outcomes for RT for LM and LMM. Common late adverse effects included hypopigmentation, teleangiectasia and skin atrophy comparable to radiotherapy series for other skin neoplasms. However, the rate of late adverse effects may be underestimated by the retrospective nature of all included studies without systematic assessment of toxicity in most cases. Late effects of radiotherapy are generally highly dependent on the specific dose and fractionation regimen as well as on the field size and localization of the target area [28]. Based on the available evidence, it has been suggested that cosmetic results in LM radiotherapy may be more favorable with Grenz rays compared to superficial X-rays [6]. Given the relatively high average age of patients with LM or LMM treated with radiotherapy, long-term adverse effects may be less relevant for the choice of the therapeutic modality.

Notably, no radiation ulcers or fibrosis were reported. Finally, the risk of second cancers induced by radiotherapy is likely to be negligible in this elderly patient population and was not reported by any study.

### Limitations

An essential limitation of our review is the low level of evidence for radiotherapy of LM and LMM. All studies using radiotherapy were retrospective and from single institutions, and the majority did not report on other treatment options such as surgery or imiquimod. Additionally, some studies included LM which was only detected clinically without biopsy, and treatment techniques as well as recurrence rates were not reported consistently. According to a recent Cochrane systematic review, data quality is a major issue for any treatment option for LM [4].

The comparison of therapeutic alternatives and their outcome for LM and LMM is mainly based on single modality patient series from different institutions [6]. Therefore, any comparisons of radiotherapy series to surgical series have to be made with caution, as patients undergoing radiotherapy often have unfavorable characteristics such as large lesions or comorbidities which may result in inferior outcome irrespective of the therapeutic modality [7].

The Australian phase 3 trial RADICAL (NCT02394132) is currently the only ongoing prospective trial on radiotherapy for LM listed on *clinicaltrials.gov*. The trial investigates the efficacy of radiotherapy versus topical imiquimod as a non-surgical treatment for biopsy-proven LM. The primary endpoint is the proportion of treatment failure 6 months after completion of the treatment. The trial is going to enroll a total of 266 patients, and the study completion date is expected in 2021. The protocol recommends normofractionated RT with single fraction doses of 2 Gy and a total dose of 54–60 Gy as the standard regimen for the definitive treatment of LM. Alternatively, hypofractionated schedules with single fraction doses of up to 4 Gy can be used.

## Conclusion

In summary, radiotherapy for LM and LMM seems to provide excellent local control with good cosmetic outcomes and is considered the preferred non-surgical treatment modality. Radiotherapy should be discussed in a multidisciplinary meeting as a treatment option particularly for elderly people with lesions in the head and neck region who have contraindications or risk factors for surgery.

As there is a lack of high-quality data and comparative evidence, the results of the currently ongoing phase 3 trial RADICAL (NCT02394132) comparing radiotherapy and topical imiquimod are eagerly awaited.

## Data Availability

The datasets used and analyzed during the current study are available from the corresponding author on reasonable request.
